# Declining trends in adolescent alcohol consumption and related harms: No room for complacency (an empirical reply to Vieira *et al*. 2025)

**DOI:** 10.1111/add.70413

**Published:** 2026-03-30

**Authors:** Charlotte R. Pennington, Daniel J. Shaw, Magda Skubera, Abigail K. Rose, Andrew Jones

**Affiliations:** ^1^ Department of Psychology Aston University Birmingham UK; ^2^ Institute of Health and Neurodevelopment Aston University Birmingham UK; ^3^ School of Psychology Liverpool John Moores University Liverpool UK

**Keywords:** adolescence, alcohol consumption, alcohol‐related harm, drinking declines, prevalence, public health, trends

## Abstract

Vieira *et al.* report that alcohol‐related harms among adolescents have generally declined in high‐income countries where youth drinking has decreased, but several methodological choices complicate this conclusion. By performing reproducibility analyses on Vieira *et al.*'s raw data, we show that their findings are more nuanced and complex. Secondary data analyses reveal that 19–24‐year‐olds have elevated vulnerability to alcohol‐related harms. Any discussion of declining trends in adolescent alcohol consumption and related harms should acknowledge that current prevalence rates and harms remain unacceptably high and require continued public health attention.

## INTRODUCTION

Vieira et al. [1] report the results of an important and timely systematic review of trends in adolescent alcohol‐related harms in high‐income countries where a decline in alcohol consumption has occurred. They conclude that “alcohol‐related harms for young people have generally declined in countries where youth drinking has fallen”. In this commentary, we highlight some methodological decisions that change several of their interpretations. First, Vieira et al. focus their review on high‐income countries identified in Vashishtha et al. where at least a 30% decrease in adolescent alcohol consumption is calculated, but we present issues of selection effects. Second, Vieira et al. report relative but not absolute risk estimates and neglect critical discussion of whether current prevalence rates of adolescent alcohol consumption and harms are *practically meaningful*. Third, they adapt their comparison date from 2020 onward to 2019 due to the COVID‐19 pandemic but do not state how this impacts their inferences. Fourth, they focus on the age range of 10–19 but some of their data extend up to 34 years of age, and data for the age category of 19–24‐year‐olds (late adolescence) is omitted. By performing reproducibility analyses on Vieira et al.’s raw data, we show that their findings are more nuanced and complex. Through secondary data analyses, we present findings for 19–24‐year‐olds, indicating that this age range confers elevated vulnerability to alcohol‐related harms. In doing so, we make the case that any discussion of declining trends in adolescent alcohol consumption and related harms should acknowledge that current prevalence rates and harms in adolescence remain unacceptably high and should remain a public health focus.

## SELECTION EFFECTS CHANGE INTERPRETATIONS

Vieira *et al*. [1] restricted their systematic review to high income countries in which Vashishtha *et al*. [2] report at least a 30% reduction in the prevalence of adolescent drinking since the early 2000s. The reason behind this methodological decision is unclear, but is perhaps reflected in the statement, ‘because these countries should have experienced the most substantial and, therefore, detectable reductions in alcohol‐related harms’ ([1], p. 1552). However, this presents an issue of range restriction/enhancement: reducing sampling variability can change the direction and magnitude of correlations [3]. Put simply, because prevalence estimates of alcohol consumption will generally correlate positively with alcohol‐related harms, selecting countries with such large prevalence reductions may bias findings on alcohol‐related harm in the same direction and further impacts generalisability. Vashishtha *et al*. [2] report an additional 16 high‐income countries in which alcohol consumption declined less than 30%, which were excluded from Vieira *et al*.’s [1] review. The finding from Vieira *et al*. [1] that ‘harms have mostly declined in line with consumption’ (p. 1566) is therefore unsurprising given this selection effect and cannot tell us anything about alcohol‐related harms for *all* high‐income countries.

Furthermore, Vashishtha *et al*. [2] calculated the percentage decline in adolescent drinking from the latest available year relative to a baseline year where prevalence was highest (‘peak year’) [2]. This accentuates declines compared to if the first available year of data is used as the baseline. In Table S1 we recalculate the percentage change based on the first available year from Vashishtha *et al*. [2], which shows that five countries would have been excluded from Vieira *et al*.’s [1] review for not meeting the threshold of a 30% decline in adolescent alcohol consumption. Our view is that different methodological inclusion criteria change Vieira *et al*.’s [1] findings: their approach maximises decline estimates, which is particularly problematic in the presence of high year‐to‐year sampling variability.

## ABSOLUTE RISK SHOWS THAT ADOLESCENT ALCOHOL CONSUMPTION AND RELATED HARMS REMAIN A CONCERN

Vieira *et al*. [1] present the percentage decline in adolescent alcohol consumption from the 19 included countries that range from 30% to 84%. These extreme numbers demonstrate a trend of substantial declines in adolescent alcohol consumption in high‐income countries over the past two decades. However, the authors do not report the baseline and comparator estimates for each time point. These absolute values are crucial to infer whether we should remain concerned about the prevalence of adolescent alcohol consumption. As shown in Table 1, the comparator estimates (time 2) range from 9% (Iceland) to 42% (Portugal), with an average of 29.5%, for past month drinking between the ages 12 to 18. Using the same baseline as Vieira *et al*. [1], 12 of 19 countries have a current prevalence rate of 30% or more. Despite declining over time, this still represents a high proportion of adolescents consuming alcohol. Furthermore, 10 of the 16 high‐income countries that were excluded from Vieira *et al*.’s [1] review have prevalence rates more than 50% 1 based on the latest available time point [2]. It is well established that early alcohol initiation and binge drinking in adolescence confer increased vulnerability for later alcohol use problems, along with neurocognitive alterations [4, 5, 6, 7, 8]. We argue that these prevalence rates are still consequential and discourage complacency: despite general declines in adolescent alcohol consumption over time, an alarmingly high percentage of adolescents still consume alcohol before the legal age in many countries [9] and this leads to wholly preventable harms. This caution is absent from Vieira *et al*. [1].

**TABLE 1:** Prevalence of adolescent past month or monthly drinking for high‐income countries included in Vieira *et al*. [1].

Country	Data source	Time period	Age, years	Percentage decline (%)	Time 1 (%)	Time 2 (%)
Australia	ASSAD	2002–2017	12–17	44.9	49	27
Austria	HBSC	2005/2006–2013/2014	11, 13 and 15	36.6	57	**36**
Belgium	HBSC	2005/2006–2013/2014	11, 13 and 15	37.5 (Flemish); 38.2 (French)	60 (Flemish); 49 (French)	**38** (Flemish); **30** (French)
Canada	HBSC	2001/2002–2013/2014	11, 13 and 15	38.4	45	28
Estonia	ESPAD	1999–2015	15	38.7	62	**38**
Finland	ESPAD	1999–2015	15	47.5	61	**32**
Germany	HBSC	2001/2002–2013/2014	11, 13 and 15	30.4	50	**35**
Iceland	ESPAD	1995–2015	15	83.9	56	9
Ireland	HBSC	1997/1998–2013/2014	11, 13 and 15	64.4	45	16
Lithuania	ESPAD	2003–2015	15	55.8	77	**34**
The Netherlands	HBSC	2005/2006–2013/2014	11, 13 and 15	41.1	55	**32**
New Zealand	Youth2000	2001–2012	12–18	43.0	55	**31**
Norway	ESPAD	1999–2015	15	60.0	55	22
Portugal	ESPAD	2007–2015	15	30.0	60	**42**
Spain	HBSC	2009/2010–2013/2014	11, 13 and 15	35.7	42	27
Sweden	ESPAD	1999–2015	15	53.6	56	26
Switzerland	HBSC	2001/2002–2013/2014	11, 13 and 15	44.9	42	23
United Kingdom	HBSC	2001/2002–2013/2014	11, 13 and 15	46.2	65	**35**
United States of America	YRBSS	1999–2017	14–18	40.4	50	**30**

*Notes*: Time 2 prevalence estimates above 30% are highlighted in bold. We have included additional information on ‘Data Source’, ‘Age’, ‘Time 1’ and ‘Time 2’ from the data reported by Vieira *et al*. [1]. Time 1, first year of decline; Time 2, latest available year.

Abbreviations: ASSAD, The Australian Secondary Students' Alcohol and Drug Survey; ESPAD, European School Survey Project on Alcohol and Drugs; HBSC, Health Behaviour in School‐Aged Children; YRBSS, Youth Risk Behaviour Survey; Youth2000, Youth2000 Health Survey.

The same issue occurs with the absence of baseline and comparison statistics for alcohol‐related harms, so we report them in Table S2. The largest decrease comes from Ireland [10], with a 90% reduction in alcohol‐related hospital discharges in 0‐ to 17‐year‐olds from 14.1% in 1995 to 1.40% in 2018. However, another record from Ireland [11] suggests an increase of 0.73% annual percentage change for alcohol‐related chronic liver disease (ALD) and cirrhosis in 15‐ to 19‐year‐olds from 2000 to 2019. This likely reflects the different age range of the two samples, highlighting the importance of delineating among narrow age ranges when reporting alcohol‐related harms. When combining countries, the same report [11] highlights that in the latest available data, there were 281 450 ALD cases, 18 930 incidences and 3190 deaths among adolescents. The largest increases were measured in Switzerland [12], with a 94% increase in the number of alcohol‐related hospital admissions from 67 to 130 2 in 10‐ to 14‐year‐olds from 2005 to 2019. The United States [13] also shows a 63% increase in the rates of emergency department presentations of alcohol‐related harms for < 20‐year‐olds, rising from 10.8 to 17.6 of 100 visits from 2009/2010 to 2014/2015. By presenting only relative change, there is a risk that readers of Vieira *et al*. [1] will perceive general declines as overly optimistic (see [14]). A decrease of 90%, for example, is dramatic, but absolute risk estimates indicate that adolescent alcohol consumption and related harms should remain a public health concern. We call for addiction researchers to situate alcohol consumption prevalence and harm estimates in their context.

## REPRODUCING ESTIMATES FROM VIEIRA *ET AL*: A MORE NUANCED PICTURE EMERGES

To recap, the primary outcome variable from Vieira *et al*. [1] was the percentage change in alcohol‐related harms between two time points: the comparator was set to 2019 because of potential changes caused thereafter by the coronavirus disease 2019 (COVID‐19) pandemic, and the initial time point started at 2005 or later, ‘reflecting the period in which youth drinking has declined’ (p. 1553). The authors decided on a 10% threshold to denote ‘meaningful change’, with percentages less than 10% deemed stable. The direction of trends are therefore reported as a ‘Decrease’, ‘Increase’, ‘Stable’, or ‘Mixed’, with the last classification used where records present any combination of the other three classifications (e.g. where sex differences were apparent).

On request, the first author (Vieira) shared the raw data of their review enabling us to perform reproducibility checks by recalculating their percentage change estimates. For each record, Vieira *et al*. [1] categorise the overall direction of trends by combining different age groups, but we report each age group separately—a decision we feel necessary because of increasing alcohol consumption and related harms from early to late adolescence [15]. Vieira *et al*. [1] report the results from *n* = 37 records, but reporting age groups separately provides a total of *n* = 47 (data available at: https://osf.io/kmpqb/). We successfully reproduced *n* = 37 (78.7%) records, partially reproduced *n* = 6 (12.8%) and were unable to reproduce *n* = 4 (8.51%). Findings that were partially reproducible comprise either minor reporting errors that do not change the interpretation of findings or reflect changes in data availability (e.g. website updates), so are not reported herein (see Table S2). The four results that could not be reproduced came from three records. The first was from the ‘Diagnostic data of the hospitals starting from 2000’ [16] 3. Vieira *et al*. [1] report ‘Mixed’ results from this record based on sex differences, but their raw data indicate that this is only accurate for 10‐ to 14‐year‐olds — both females and males in the 15 to 19 age range instead show an Increase. The second record was the ‘Yearbook of alcohol and drug statistics’ [17, 18]. Vieira *et al*. [1] report a Stable trend for < 14‐year‐olds and a Decrease for 15‐ to 19‐year‐olds in table 6 of their published article, but we compute the opposite: there was a Decrease of 26% for < 14‐year‐olds and Stable trend (−3.85%) for 15‐ to 19‐year‐olds. The third record was Green *et al*. [19]. Vieira *et al*. [1] state a ‘Decrease’ for females 15 to 19 years old, but the Lexi plots from this report suggest an increasing trend, meaning that this should have been categorised as ‘Mixed’ because of sex differences.

In their main text, Vieira *et al*. [1] state that seven records from North America showed either Decrease or Stable trends, one showed Increase and three showed Mixed trends based on sex differences (p. 1560). Table 6 (p. 1561) reports a slightly different pattern, however, and one that aligns with our reproducibility checks: *six* showed either Decrease or Stable trends, one showed an Increase and *four* showed Mixed trends. Moreover, when we distinguish Decrease from Stable trends, a more nuanced pattern emerges: four records show Decrease trends and two show Stable trends. We were able to reproduce the findings reported by Vieira *et al*. [1] for all records from other Anglosphere countries and mainland Europe. In summary, across high‐income countries included in their review, there are 16 records of Decrease, 12 Mixed, five Stable and four Increase trends. Although this does not change the overall interpretation that ‘harms have mostly declined in line with consumption’, we feel it is important to highlight these discrepancies for independent teams inferring from or re‐using the data.

When reporting sex differences, Vieira *et al*. [1] report that for North America, three of four records showed Increase trends and one a Decrease for females, which we reproduced. We note a discrepancy for males, however: the authors report that three records indicate a Decrease and one record indicates a Stable trend (p. 1560), yet their table 6 states that *two* records indicate a Decrease and *two* indicate Stable trends. Our reproducibility check suggests that it is table 6 which is correct. For mainland Europe, Vieira *et al*. [1] report a total of four sex‐specific differences in the main text (p. 1565), but we identify *five* in table 6. Three of these records indicate Decrease for both females and males, one Increase for both females and males and one Mixed trend based on a sex × age interaction — specifically, a Decrease for males but an Increase for females at 10 to 14 years, and an Increase for both males and females at 15 to 19 years. We reproduce the analyses for Anglosphere countries. Across these records, then, there are more Decrease trends for males (*n* = 13) compared to females (*n* = 8), comparable Stable trends for males (*n* = 2) and females (*n* = 3) and more Increase trends for females (*n* = 6) than males (*n* = 2). It is important to note that further exploration of this data, where available, suggests that this is among a backdrop of males showing higher harms than females in seven records, females showing higher harms than males in six records and comparable harms in two records. Together, this suggests that there are unique populations of alcohol‐related harms. Although decreases have mainly been seen for males and females, there are more records indicating an increase for young adolescent females than males. A future research direction is to ascertain explanations for such sex differences [20].

A final word on reproducibility, the article's Data Availability Statement states ‘Data sharing is not applicable to this article as no new data were created’, but new data was created—the percentage change scores calculated from each time point. After contacting the first author, they kindly shared their (clear and transparent) data, but we would not have been able to conduct reproducibility checks without this. We recommend that addiction researchers share their data on public repositories wherever possible (‘as open as possible, as closed as necessary’) [21] to enhance the reproducibility and credibility of this field [22]. The positive consequences of data sharing are highlighted here—we now conduct a secondary data analysis of Vieira *et al*. [1] made possible through their sharing of raw data.

## SECONDARY DATA ANALYSIS 1: (RE)DEFINING ADOLESCENCE REVEALS THAT HARMS HAVE GENERALLY INCREASED FOR 19‐ TO 24‐YEAR‐OLDS

Vieira *et al*. [1] state that they reviewed data for ‘adolescents aged between 10‐ and 19‐years old, either as an age group within this range or as a young adult group up to the age of 34 that overlaps with this range (e.g. age groups of 18–24 or 15–34 included)’ (p. 1552). We note three limitations to this approach: (1) the upper age limit of 34 extends beyond their focus on adolescence; (2) the specific age range of 19 to 24 years was not fully reviewed, despite many scholars considering adolescence to encompass this range; and (3) where a record within the age range of 10 to 19 years was identified (e.g. age 12–17 years), other records in their expanded range (e.g. age 18–24 years) were not reviewed even though ages did not overlap.

Although Vieira *et al*.’s [1] rationale for focusing on the age range of 10 to 19 is not stated explicitly, it aligns with the World Health Organisation's [23] definition of adolescence. A wealth of developmental neuroscience research suggests, however, that the period of adolescence extends to 24 years [24, 25], and this more inclusive definition is essential for developmentally appropriate framing of laws, social policies and service systems [26]. During this protracted critical developmental window, the brain undergoes profound maturational events in both structure and function that makes adolescents particularly vulnerable to the effects of alcohol [6, 7, 27]. Further, the risks of alcohol‐related harm in later adolescence may be influenced by the availability (legality) of alcohol in high income countries. Indeed, a recent meta‐analysis indicates that alcohol consumption peaks in later adolescence [28] and a recent assessment of 204 countries indicates that alcohol use is a leading risk factor for the attributable burden of disease among those 10 to 24 years old [29, 30].

Based on this rationale, we performed a secondary data analysis on the records reviewed by Vieira *et al*. [1] to expand the upper age limit from 19 to 24 years. We identified four records that were excluded from Vieira *et al*. [1] because of the third issue outlined above: two from the 15 to 34 age range [31], one from the 18 to 24 age range [32] and one from the 14 to 29 age range [33]. In total, we identified an additional 18 datasets from the same main records reviewed by Vieira *et al*. [1], which span the ages 19 to 24 years. This revealed mainly *Increase* trends (*n* = 7), followed by Mixed (*n* = 5, based on sex or country differences), Decrease (*n* = 4) and Stable trends (*n* = 2) (Table 2). When adding these to the *n* = 37 records reviewed by Vieira *et al*. [1], we yield a total of *n* = 55, with 20 records showing Decrease trends, 17 Mixed, 11 Increase and seven Stable trends for adolescents aged 10 to 24 years old. In North America, four records show Mixed trends, three Increases, one Stable and one Decrease trends. In other Anglosphere countries, there are three Mixed trends, three Decreases and two Increases. In Europe, three records show a Decrease, two Increases, two Mixed and one Stable. A total of eight records provided data on sex differences indicating that males (*n* = 5) *and* females mainly showed *increasing* alcohol‐related harms (*n* = 6). As such, when focusing on the age range of 18 to 24 years old specifically, trends in alcohol‐related harms have generally increased.

**TABLE 2:** Key results from a secondary data analysis focusing on late adolescence (*n* = 25).

Country	First author, publication year OR data summary	Direction of trend	Age, years	Proportion	Timespan	Measure	Notes
North America (*n* = 9)							
US	Moise, 2019	Decrease	20–24	23% decrease: 22.2 to 17.1 per 100 000	Comparing 2006–2010 to 2011–2015	Age‐standardised rates of alcohol‐positive unintentional injury hospitalisations	
US	White *et al*. [32], 2018	Increase	18–24	APC of 2.4 (*P* < 0.01)	2006–2014	Rates of all alcohol‐related ED visits per 100 000 population	
US	Chen and Yoon, 2018 (‘Trends in alcohol‐related morbidity among community hospital discharges)	Stable	21–24	Stable (8.30% increase): 3.5 to 3.7 per 10 000.	2005–2015	Principal alcohol‐related discharges per 10 000 population	Comparison statistic of 1103.9 per 100 000 but no baseline statistic
Canada	Canadian substance use costs and harms	Increase	15–34	37.50% increase: 3422 to 4705 per 100 000	2007–2019	Unstandardized rates of alcohol‐attributable ED visits per 100 000	
Canada	Canadian substance use costs and harms	Mixed	15–34	19.54% increase overall: 138.73 to 165.84 per 100 000 Stable (6.60% increase) for males: 195.89 to 208.83 50.5% increase for females: 79.97 to 120.36	2007–2019	Unstandardized rates of alcohol‐attributable hospitalisations per 100 000	Categorised as ‘Mixed’ because of sex differences
US	Ngo *et al*. [13], 2018	Increase	20 to <25	25% increase: 7.2 to 9 of 100 visits	2009/2010–2014/2015	Rates of alcohol‐related ED presentations per 100 ED visits	
Canada	Smith *et al*., 2023	Mixed	15–34	Stable (3.5% increase) in rates for males: 170 to 176 per 100 000 43% increase for females: 60 to 86 per 100 000	2008–2019	Rates of alcohol‐attributable hospitalisations per 100 000 population	Already reported by Vieira *et al*. [1]
Canada	Smith *et al*., 2023	Mixed	15–34	16% decrease in rates for 15‐ to 34‐year‐old males: 3603 to 3011 per 100 000 17% increase in rates for 15‐ to 34‐year‐old females: 1328 to 1550 per 100 000	2008–2019	Rates of alcohol‐attributable ED visits per 100 000 population	Already reported by Vieira *et al*. [1]
North America	Danpanichkul *et al*. [11], 2024	Mixed	20–24	Stable APC for Canada (0.52) and Decrease for US (−3.03)	2000–2019	APC for alcohol‐related cases of chronic liver disease and cirrhosis	Categorised as ‘Mixed’ because of country differences
Other Anglosphere countries (*n* = 8)							
UK: England	Green *et al*. [19], 2017	Increase	20–24	Increasing trends for both males and females: ~70–80 to 150 per 100 000	2005/2006–2013/2014	Rates of acute hospital admissions wholly attributable to alcohol per 100 000 population	Used same method as Vieira *et al*. [1] by approximating based on Lexi plots
Australia	Sims *et al*., 2020	Decrease	12–24	Alcohol‐related hospitalisations increase 0.5% per year but decrease to −1.7% when matched with hospitalisations. APC of −6.2 for 12‐ to 14‐year‐olds (−5.8 for males and −6.6 for females) APC of −4.2 for 15‐ to 17‐year‐olds (−4.6 for males and −3.7 for females)	2005/2006–2016/2017	Rates of alcohol‐related ED presentations (after matching with subsequent hospitalisations) per 10 000 population	Already reported by Vieira *et al*. [1]
New Zealand	Alcohol‐related harm data	Decrease	15–24	10.68% decrease: 32.30 to 28.85 per 100 000	2005–2019	Rates of hospitalisations wholly attributable to alcohol per 100 000	Already reported by Vieira *et al*. [1]
UK: Scotland	Alcohol related hospital statistics	Decrease	20–24	33% decrease: 589.2 to 394.8 per 100 000 40% decrease for males: 828 to 494.4 16% decrease for females: 350.5 to 295.2	2005/2006–2018/2019	Rates of alcohol‐related hospital admissions per 100 000 population	
UK: England	Tyrrell *et al*., 2018	Increase	19–24	Males appear to have increased from ~90 to >150 (~67%) 117% increase for females: 85.4 to 185.2 per 100 000	1998–2014	Rates of alcohol‐related poisoning events per 100 000 population	Data for males only presented in figure 2 of Tyrrell *et al.* 2018 so approximates stated
Australia	Australian alcohol‐attributable harm visualisation tool	Mixed	15–34	Stable trend (9%): 208.93 to 190.89 per 100 000 14% decrease for males: 245.33 to 210.90 Stable (0.58% decrease) for females: 171.33 to 170.33	2011–2019	Age‐specific rates of alcohol‐attributable hospitalisations per 100 000 population	Categorised as ‘Mixed’ because of sex differences
Australia	Alcohol‐related injury: hospitalisations and deaths	Mixed	15–24	21% decrease: 6272 to 4970 per 100 000 29% decrease for males: 4152 to 2120 Stable (5.14% decrease) for females: 2959 to 2011	2010/2011–2016/2017	No. of alcohol‐related injury hospitalisations	Already reported by Vieira *et al*. [1]
Other Anglosphere countries	Danpanichkul *et al*. [11], 2024	Mixed	20–24	Stable APC for Australia (−0.24), Increase for Ireland (0.79), Stable for New Zealand (0.08) and Stable for UK (−0.07)	2000–2019	APC for alcohol‐related cases of chronic liver disease and cirrhosis	Mixed based on country differences
Mainland Europe (*n* = 8)							
Sweden	Statistical database, diagnoses	Mixed	20–24	Stable (8.5% decrease): 526.9 to 482.0 per 100 000 11.61% decrease for males: 547.8 to 484.2 Stable for females (5.03% decrease): 505.1 to 479.7	2008–2019	Age‐standardised rates of alcohol‐related in‐patient or specialised open care (ED or hospital admissions) per 100 000 population	Categorised as ‘Mixed’ because of sex differences
Spain	Mortality attributable to alcohol in Spain	Decrease	15–34	70% decrease: excessive drinkers: 70% decrease from 3.3 to 1.0 per 100 000 light/moderate drinkers: 56% decrease from 3.2 to 1.4 per 100 000	Comparing 2001–2009 to 2010–2017	Mortality rates attributable to alcohol per 100 000 population	Already reported by Vieira *et al*. [1]
Lithuania	Prevalence—number of ill people	Decrease	14–29	12% decrease: 4.96 to 4.35 per 1000 population	2014–2019	Rates of alcohol‐related diagnoses in healthcare institutions and on death certificates, per 1000	
Switzerland	Patients in hospitals by age, class, sex and diagnostic group (Patients dans les hôpitaux selon la classe l'âge, le sexe et le groupe de diagnostic)	Increase	20–24	76% increase: 289 to 509	2005–2019	No. of alcohol‐related hospital admissions	No denominator provided
Germany	Diagnostic data of the hospitals starting from 2000	Increase	20–25	15.2% increase for males: 5043 to 5811 per 100 000 28.7% increase for females: 2348 to 3023	2005–2019	Rates of hospital discharges for alcohol‐related diagnoses per 100 000 inhabitants	
Switzerland	Wicki, 2020	Stable	20–24	Stable (9.5% increase): 34.9 to 38.2 per 1000	2010–2016	Proportion rates of alcohol‐related hospitalisations when accounting for Switzerland and excluding the Canton of Vaud (area of alcohol policy changes)	Below ‘meaningful’ 10% threshold so ‘Stable’
Finland	Yearbook of Alcohol and Drug Statistics (Päihdetilastollinen vuosikirja; Alkoholi ja huumeet)	Decrease	19–25	−29.40% decrease: 452 to 319 per 100 000	2005–2019	No. of hospital inpatient care periods with a primary diagnosis of alcohol per 100 000	
Mainland Europe	Danpanichkul *et al*. [11], 2024	Mixed	20–24	Decrease APC for Austria (−1.13), Stable for Belgium (0.01), Increase for Estonia (1.09), Stable for Finland (0.37), Stable for Germany (−0.40), Increase for Iceland (0.69), Increase for Lithuania (1.42), Stable for Netherlands (0.11), Increase for Norway (0.81), Decrease for Portugal (−2.96), Decrease for Spain (−1.70), Decrease for Sweden (−0.97), Stable for Switzerland (0.27)	2000–2019	APC for alcohol‐related cases of chronic liver disease and cirrhosis	

*Note*: Seven records were already included in Vieira *et al*. [1], and we identified 18 additional datasets from the same records included in their review. All references from this table can be found in the supplementary materials.

Abbreviations: APC, annual percentage change; ED, emergency department; UK, United Kingdom; US, United States;

Another key finding from our secondary analyses is that alcohol‐related harms increase with age during adolescence. To exemplify from records that provide directly comparative data on different age categories, one report [32] states that for every 100 000 alcohol‐related emergency department visits, 228.1 were for 12‐ to 17‐year‐olds, but this increases fivefold to 1103.9 for 18‐ to 24‐year‐olds. In a second report [31], the rate of alcohol‐attributable hospital and emergency department admissions increases from 13.12 per 100 000 for 0‐ to 14‐year‐olds to 4705 for 15‐ to 34‐year‐olds. In a third [16], the rate of 1076 per 100 000 inpatient discharges for 10‐ to 14‐year‐olds increases dramatically to 8834 for 20‐ to 25‐year‐olds. This suggests that late adolescence represents a developmental stage of heightened vulnerability to alcohol‐related harms. Monitoring these trends in subpopulations of narrower age ranges of adolescence that together span more years is crucial to inform equitable prevention and intervention strategies.

## SECONDARY DATA ANALYSIS 2: EXPLORING WHETHER PREREGISTRATION DEVIATIONS INFLUENCE INFERENCES

In their published article, Vieira *et al*. [1] state that they ‘did not include data from 2020 onward to reduce the potential impact of coronavirus disease 2019 on our results’ (p. 1553). Although this appears to be a reasonable and defensible methodological decision, inspection of the raw datafile reveals that this decision was made after pre‐registering the study protocol [34] and details that for some records, ‘This also updated/changed the direction of results’. These changes are not reported in the final article and, therefore, in the absence of updates to the pre‐registration and publicly available raw data, remain unknown. Furthermore, one record that spanned dates encompassing the COVID‐19 pandemic was included [35] because such data was only available for an ‘entire period’.

It is, nonetheless, important to understand current alcohol consumption prevalence and related harms in adolescence. We therefore conducted an additional secondary data analysis to explore the potential influence of this deviation. Vieira *et al*.’s [1] raw data file identifies nine records affected by this decision, increasing to 12 when we report age groups separately. With the adapted cut‐off date of 2019, six of these show Decrease trends, three Increases, two Stable and one Mixed based on sex differences. Reverting this to the latest available date, which spans up to the year 2022, changes the results for two records: one Increase becomes a Decrease, and one Increase becomes Mixed based on sex differences (Table S3).

Furthermore, Vieira *et al*. [1] exclude nine records from the main review that cover a timespan of less than 5 years, which are reported separately in their supplementary materials. Their pre‐registration does not state this as an exclusion criterion, but does suggest that such studies would be considered less robust than those with longer time periods. In a risk assessment, however, the authors categorised six of these records as low risk and three as moderate risk, which is similar to the included studies spanning more than 5 years. These nine records report five Increase trends, two Stable, one Decrease and one Mixed trend. Including these records changes the findings slightly to: 17 records of Decrease trends, 13 Mixed, nine Increases and seven Stable trends.

Both methodological decisions can be justified and do not substantially alter Vieira *et al*.’s [1] main conclusions, but our point is that apparent deviations are not labelled as such. Crucially, then, the reader has no way of identifying these without looking at the raw data (which are not publicly shared) and/or assessing pre‐registration‐manuscript consistency. Indeed, deviations may be appropriate, but they must be reported transparently and their impact on inferences should be discussed—whether influential or not [36].

## CONCLUSION

The systematic review by Vieira *et al*. [1] asks an essential and timely question: what are the trends in alcohol‐related harms for young people in countries where drinking has substantially declined? However, we posit that methodological choices and the reporting of findings impact the answer to this question. We highlight that their approach maximises decline estimates, does not acknowledge absolute risk, and captures only part of late adolescence. Focusing on relative declines alone lacks contextualisation and may encourage misplaced reassurance when absolute alcohol consumption prevalence and harm levels remain high.

We argue that estimates are practically meaningful. Consumption prevalence remains alarmingly high across the high‐income countries included in Vieira *et al*.’s [1] review and certain populations appear to be particularly vulnerable to alcohol‐related harms, specifically young females and adolescents aged 18 to 24 years.

The danger is this: if policymakers, funders and stakeholders conclude that alcohol consumption and related harms are dramatically declining for adolescents, there is a risk that funding for treatment and research will not be prioritised and support for adolescents and young people will be further displaced. Research on adolescent alcohol consumption should be recognised as a key public health focus to ensure that appropriate policies, prevention, intervention and harm reduction approaches are implemented.

## AUTHOR CONTRIBUTIONS

**Charlotte R. Pennington:** Conceptualization (lead); data curation (lead); formal analysis (lead); funding acquisition (equal); investigation (lead); methodology (lead); project administration (lead); resources (lead); software (lead); supervision (lead); validation (lead); writing—original draft (lead); writing—review and editing (lead). **Daniel J. Shaw:** Conceptualization (supporting); writing—original draft (equal); writing—review and editing (supporting). **Magda Skubera:** Investigation (supporting); validation (supporting); writing—review and editing (supporting). **Abigail K. Rose:** Writing—review and editing (supporting). **Andrew Jones:** Conceptualization (supporting); investigation (supporting); validation (supporting); writing—original draft (supporting); writing—review and editing (equal).

## DECLARATION OF INTERESTS

None.

## Supporting information


**Table S1.**Prevalence of adolescent past month or monthly drinking for high‐income countries reproduced from Vashishtha et al. (2020). Here, we calculate percentage decline between the earliest timepoint to the most recent year available (rather than between the “peak year” to the most recent year available as per Vashishtha et al.). Any changes from Vashishtha et al. (and therefore Vieira et al.) are highlighted in **bold** and an asterisk denotes when this falls below the inclusion criteria of a 30% decline.
**Table S2.** Reproducibility checks of Vieira et al. (2025). Any changes are highlighted in **bold** in the relevant column. Note that Vieira et al. report data for different age groups for each record in a single cell whereas we report them individually. The total number of included studies in Vieira et al. is N = 37, and with age groups reported individually this becomes N = 47. Data are available at: https://osf.io/kmpqb/. All references are cited in Vieira et al.
**Table S3.** Secondary data analysis of records with a comparison timepoint within the years of 2020–‐2022 – the years stated as the comparison in Vieira et al.’s PROSPERO registration but not adopted in their final analysis. Any changes are highlighted in **bold** in the relevant column. All references are cited in Vieira et al.

## Data Availability

The raw data associated with this article is publicly available via the Open Science Framework in the folder ‘Data’: https://osf.io/kmpqb/.

## Footnotes

^1^
Prevalence rates for these 10 countries are as follows: Denmark (75%), Cyprus (68%), Greece (66%), Italy (57%), Malta (54%), Monaco (54%), Czech Republic (68%), Hungary (55%), Latvia (65%) and Slovenia (52%).

^2^
No denominator values are provided.

^3^
Vieira *et al*. [1] state that they had difficulty retrieving the data from this report and we ran into the same issue. We, therefore, use Vieira *et al*.’s [1] raw data in this case.
