# Supplementary material for: Declining trends in adolescent alcohol consumption and related harms: No room for complacency (an empirical reply to Vieira et al. 2025)
Source: Addiction. 2026 Mar 30;121(8):2252–61. doi: 10.1111/add.70413 (PMC13357884; doi:10.1111/add.70413)
Supplement: Supplementary file 1 — Table S1.Prevalence of adolescent past month or monthly drinking for high‐income countries reproduced from Vashishtha et al. (2020). Here, we calculate percentage decline between the earliest timepoint to the most recent year available (rather than between the “peak year” to the most recent year available as per Vashishtha et al.). Any changes from Vashishtha et al. (and therefore Vieira et al.) are highlighted in bold and an asterisk denotes when this falls below the inclusion criteria of a 30% decline. Table S2. Reproducibility checks of Vieira et al. (2025). Any changes are highlighted in bold in the relevant column. Note that Vieira et al. report data for different age groups for each record in a single cell whereas we report them individually. The total number of included studies in Vieira et al. is N = 37, and with age groups reported individually this becomes N = 47. Data are available at: https://osf.io/kmpqb/. All references are cited in Vieira et al. Table S3. Secondary data analysis of records with a comparison timepoint within the years of 2020–‐2022 – the years stated as the comparison in Vieira et al.’s PROSPERO registration but not adopted in their final analysis. Any changes are highlighted in bold in the relevant column. All references are cited in Vieira et al. [file ADD-121-2252-s001.docx]

**Declining trends in adolescent alcohol consumption and related harms: No room for complacency (an empirical reply to Vieira et al. 2025)**

**Charlotte R. Pennington*^1,2^, Daniel J. Shaw^1,2^, Magda Skubera^1^, Abigail K. Rose^3^ &, Andrew Jones^3^**

**Supplementary Materials**

**Table S1.** Prevalence of adolescent past month or monthly drinking for high-income countries reproduced from Vashishtha et al. (2020). Here, we calculate percentage decline between the earliest timepoint to the most recent year available (rather than between the “peak year” to the most recent year available as per Vashishtha et al.). Any changes from Vashishtha et al. (and therefore Vieira et al.) are highlighted in **bold** and an asterisk denotes when this falls below the inclusion criteria of a 30% decline.

| Country | Data source | Time period | Age | Percentage decline (%) | Time 1 (%) | Time 2 (%) |
| --- | --- | --- | --- | --- | --- | --- |
| Australia | ASSAD | 1997/1998-2017 | 12 to 17 | 44.9 | 49 | 27 |
| Austria | HBSC | 2001/2002-2013/2014 | 11, 13 and 15 | **16.28*** | **43** | 36 |
| Belgium | HBSC | 2001/2002-2013/2014 | 11, 13 and 15 | **24***(Flemish); **18.92***(French) | **50**  **37** | 38  30 |
| Canada | HBSC | 1997/1998-2013/2014 | 11, 13 and 15 | **34.88** | **43** | 28 |
| Estonia | ESPAD | 1995-2015 | 15 | **25.49*** | **51** | 38 |
| Finland | ESPAD | 1995-2015 | 15 | **44.83** | **58** | 32 |
| Germany | HBSC | 2001/2002-2013/2014 | 11, 13 and 15 | 30.4 | 50 | 35 |
| Iceland | ESPAD | 1995-2015 | 15 | 83.9 | 56 | 9 |
| Ireland | HBSC | 1997/1998-2013/2014 | 11, 13 and 15 | 64.4 | 45 | 16 |
| Lithuania | ESPAD | 1995-2015 | 15 | **42.37** | **59** | 34 |
| The Netherlands | HBSC | 2001/2002-2013/2014 | 11, 13 and 15 | **30.43** | **46** | 32 |
| New Zealand | Youth2000 | 2001-2002 -2012/2013 | 12 to 18 | 43.0 | 55 | 31 |
| Norway | ESPAD | 1995-2015 | 15 | **48.84** | **43** | 22 |
| Portugal | ESPAD | 1995-2015 | 15 | **14.29*** | **49** | 42 |
| Spain | HBSC | 2003-2015 | 11, 13 and 15 | **22.86*** | **35** | 27 |
| Sweden | ESPAD | 1995-2015 | 15 | **52.73** | **55** | 26 |
| Switzerland | HBSC | 2001/2002-2013/2014 | 11, 13 and 15 | 44.9 | 42 | 23 |
| United Kingdom | HBSC | 1997/1998-2013/2014 | 11, 13 and 15 | **40.68** | **59** | 35 |
| United States of America | YRBSS | 1999-2017 | 14 to 18 | 40.4 | 50 | 30 |

**Note**: Reference: Vashishtha R, Pennay A, Dietze P, Marzan MB, Room R, Livingston M. Trends in adolescent drinking across 39 high-income countries: exploring the timing and magnitude of decline. Eur J Public Health. 2021;31(2):424–31. <https://doi.org/10.1093/eurpub/ckaa193>

**Table S2.** Reproducibility checks of Vieira et al. (2025). Any changes are highlighted in **bold** in the relevant column. Note that Vieira et al. report data for different age groups for each record in a single cell whereas we report them individually. The total number of included studies in Vieira et al. is N = 37, and with age groups reported individually this becomes N = 47. Data are available at: <https://osf.io/kmpqb/>. All references are cited in Vieira et al.

| **Country** | **First author, publication year OR data summary** | **Direction of trend** | **Age** | **Proportion** | **Timespan** | **Measure** | **Reproducibility Check** | **Reproducibility Notes** |
| --- | --- | --- | --- | --- | --- | --- | --- | --- |
| ***North America*** | | | | | | | | |
| US | Moise, 2019 | Decrease | 10-14 | 56% decrease: 0.41 to 0.18 per 100,000 | Comparing 2006-2010 to 2011-2015 | Age-standardised rates of  alcohol-positive unintentional  injury hospitalisations | YES | N/A |
| US | Moise, 2019 | Decrease | 15-19 | **38.53% decrease: 9.24 in 2006 to 5.68 per 100,000 in 2015** | Comparing 2006-2010 to 2011-2015 | Age-standardised rates of  alcohol-positive unintentional  injury hospitalisations | YES (partial) | Vieira et al. report a 36% change, but our calculation is 38.53%. However, this doesn’t change the direction or interpretation. |
| US | White, 2018 | Decrease | 12-17 | Decrease in APC of -2.7 | 2006-2014 | Rates of all alcohol-related ED  visits per 100,000 population | YES | N/A |
| Canada | Chen & Yoon, 2018 (“Trends in  alcohol-related morbidity among  community hospital discharges”) | Decrease | 12-20 | 39% decrease: 1.31 to 0.80 per 10,000 | 2005-2015 | Principal alcohol-related  discharges for 12- to 20-year-olds  per 10,000 population | YES | N/A |
| Canada | Canadian substance use costs and harms | Decrease | 0-14 | 59% decrease for 0–14-year-olds: 31.64 to 13.12 per 100,000  28% decrease for males: 16.98 to 12.21 per 100,000  70% decrease for females: 46.71 to 14.08 per 100,000 | 2007-2019 | Unstandardized rates of alcohol-attributable  ED visits per  100,000 | YES | N/A |
| Canada | Canadian substance use costs and harms | Stable | 0-14 | Stable (6% increase) in rates for 0- to  14-year-olds: 20.75 to 21.96 per 100,000 | 2007-2019 | Unstandardized rates of alcohol-attributable  hospitalisations per  100,000. | YES | N/A |
| US | National estimates of drug-related emergency department visits, 2004-2011 | Stable | 12-17 | Stable (3% decrease): 165 to 160 per 100,000 | 2005-2011 | Estimated rates of alcohol-related  ED visits per 100,000  population | YES | N/A |
| US | National estimates of drug-related emergency department visits, 2004-2011 | Stable | 18-20 | Stable (7.55% increase): 530 to 570 per 100,000 | 2005-2011 | Estimated rates of alcohol-related  ED visits per 100,000  population | YES | N/A |
| US | National estimates of drug-related emergency department visits, 2004-2011 | Stable | <21 | Stable (5.47% increase): 128 to 135 per 100,000 | 2005-2011 | Estimated rates of alcohol-related  ED visits per 100,000  population | YES | N/A |
| US | Ngo et al., 2018 | Increase | <20 | 63% increase: 10.8 to 17.6 per 100 visits | Comparing 2009/2010 to 2014/2015 | Rates of alcohol-related ED  presentations per 100 ED visits | YES | N/A |
| Canada | Smith et al., 2023 | Mixed | 15-34 | Stable (3.5% increase) in rates for males: 170 to 176 per 100,000  43% increase for females:  60 to 86 per 100,000 | 2008-2019 | Rates of alcohol-attributable  hospitalisations per 100,000  population | YES | Categorised as Mixed overall by Vieira et al. due to increase for females but stable for males. |
| Canada | Smith et al., 2023 | Mixed | 15-34 | 16% decrease in rates for 15-  to 34-year-old males: 3603 to 3011 per 100,000  17% increase in rates for 15-  to 34-year-old females: 1328 to 1550 per 100,000 | 2008-2019 | Rates of alcohol-attributable ED  visits per 100,000 population | YES | Categorised as Mixed overall by Vieira et al. because of the increase for females but decrease for males. |
| Canada | Myran et al., 2019 | Mixed | 10-18 | Stable (0.16% decrease) for males: 33.35 to 33.2 per 100,000  21% increase for females: 32.47 to 39.25 per 100,000 | 2005-2016 | Rates of alcohol-related ED  visits per 100,000 population | YES | Categorised as Mixed overall by Vieira et al. because of the increase for females but decrease for males. |
| North America | Danpanichkul et al., 2024 | Mixed | 15-19 | Stable APC (decrease of -1.85%) | 2000-2019 | APC for alcohol-related cases of  chronic liver disease and  cirrhosis | YES | N/A |
| ***Other Anglosphere countries*** | | | | | | | | |
| UK: Wales | Trefan et al., 2019 | Decrease | 10-17 | 38% decrease: 12.9 to 8.1 per 1000 | 2006-2011 | Rates of alcohol-related hospital  admissions per 1000 population | YES | N/A |
| UK: England | Green et al., 2017 | Decrease | 10-14 | Decreasing trend for males from roughly 50 to less than 50  Decreasing trend for females from roughly 100 to 50 | Comparing 2005/2006 to 2013/2014 | Rates of acute hospital  admissions wholly attributable  to alcohol per 100,000  population | YES | Approximates can be pulled from Figure 3 of the article based on heatmaps. Unclear why this was included/ categorised as a ‘Decrease’ without the raw data to calculate the percentage change. |
| UK: England | Green et al., 2017 | **Mixed** | 15-19 | Stable trend for males  Increasing trend for females | Comparing 2005/2006 to 2013/2014 | Rates of acute hospital  admissions wholly attributable  to alcohol per 100,000  population | NO | **Vieira et al. state that no raw data is provided but approximates can be pulled from Figure 3 of the article based on heatmaps. The trend for females would surpass the 10% threshold. The record is therefore ‘Mixed’.** |
| UK: England | O'Donnell et al., 2017 | Increase | 13-17 | 29% decrease: 9.1 to 6.5 per 10,000 | 1997-2012 | Rates of alcohol-related  admissions per 10,000  population | YES | N/A |
| Australia | Sims et al., 2020 | Decrease | 12-24 | Alcohol-related hospitalisations increase  0.5% per year but decrease to -1.7% when matched with hospitalisations.  APC of −6.2 for 12- to  14-year-olds (−5.8 for males  and −6.6 for females)  APC of −4.2 for 15- to  17-year-olds. (−4.6 for males  and −3.7 for females) | Comparing 2005/2006 to 2016/2017 | Rates of alcohol-related ED  presentations (after matching  with subsequent  hospitalisations) per 10,000  population | YES | N/A |
| New Zealand | Alcohol-related harm data | Decrease | 0-14 | 73% decrease: 6.18 to 1.67 per 100,000 | 2005-2019 | Rates of hospitalisations wholly  attributable to alcohol per  100,000 | YES | N/A |
| New Zealand | Alcohol-related harm data | Decrease | 15-24 | 10.68% decrease: 32.30 to 28.85 per 100,000 | 2005-2019 | Rates of hospitalisations wholly  attributable to alcohol per  100,000 | YES | N/A |
| UK: England | Local alcohol profiles for England | Decrease | <18 | 56% decrease: Vieira et al. state 24,127 to 11,233 cases  58% decrease for male  55%  decrease for females | Comparing 2006/2007 to 2018/2019 | Rates of hospital admissions  wholly attributable to alcohol  per 100,000 population | YES (partial) | Data now only goes back to 2012/13 so we could not retrieve the baseline to reproduce fully – could only go off raw data provided by Vieira et al. |
| UK: Scotland | Alcohol related hospital statistics | Decrease | <15 | 55% decrease: 42.4 to 19.1 per 100,000  57% decrease for males (39.3 to 17.0), 54%  decrease for females (45.5 to 20.9) | Comparing 2005/2006 to 2018/2019 | Rates of alcohol-related hospital  admissions per 100,000  population | YES | N/A |
| UK: Scotland | Alcohol related hospital statistics | Decrease | 15-19 | **32.88% decrease: 481.5 to 339.2 per 100,000**  **32% decrease for males: 561.8 to 385.1**  **27%**  **decrease for females: 401.2 to 293.2** | Comparing 2005/2006 to 2018/2019 | Rates of alcohol-related hospital  admissions per 100,000  population | YES (partial) | Vieira et al. report 339.2 for 2018/19 but it appears to be 323.2. However, this doesn’t change the direction or interpretation. |
| Ireland | Alcohol consumption, alcohol-related harm and alcohol policy in Ireland | Decrease | 0-17 | 90% decrease: 14.1% of discharges to 1.40%  73% decrease for males: 4.1% to 1.1% of discharges  73% decrease for females: 10% to 2.7% of discharges | 1995-2018 | Proportion of the 0- to 17-yearold  age group making up the  total amount of hospital  discharges wholly attributable  to alcohol | YES | N/A |
| Australia | Mitra et al. (2023) | Stable | 16-19 | Decrease: Incident rate ratio (IRR) of 0.95 (5% lesser likelihood from 2008-2019) | 2008-2019 | Rates of alcohol-positive  presentations to an ED trauma  centre | YES | No raw data reported only IRR. |
| UK: England | Tyrrell et al. (2018) | Mixed | 10-14 | 67% decrease for males: 94.4 to 31.2 per 100,000 for males  Unclear for females | 1998-2014 | Rates of alcohol-related  poisoning events per 100,000  population | YES | N/A |
| Australia | Australian alcohol-attributable harm visualisation tool | Mixed (based on sex differences) | 15-34 | Stable trend (9%): 208.93 to 190.89 per 100,000  14% decrease in rates for males: 245.33 to 210.90  Stable (0.58% decrease) for  females: 171.33 to 170.33 | 2011-2019 | Age-specific rates of alcohol-attributable  hospitalisations per 100,000 population | YES | N/A |
| Australia | Alcohol-related injury: hospitalisations and deaths, 2019-2020 | Mixed (based on sex differences) | 15-24 | 21% decrease: 6272 to 4970 per 100,000  29% decrease for males: 4152 to 2120  Stable (5.14%) for females: 2959 to 2011 | Comparing 2010/2011 to 2016/2017 | No. of alcohol-related injury  hospitalisations | YES (partial) | Raw data could not be identified via the link so had to rely on Vieira et al.’s raw data to compute percentage change. |
| Other Anglosphere countries | Danpanichkul et al., 2024 | Mixed | 15-19 | Stable APC for Australia (-0.14),  New Zealand (0.01) and the United  Kingdom (0.07)  Increase in APC (0.73%) for  Ireland (P < 0.001) | 2000-2019 | APC for alcohol-related cases of  chronic liver disease and  cirrhosis | YES | N/A |
| **Mainland Europe** | | | | | | | | |
| Sweden | Statistical database, diagnoses | Decrease | 10-19 | **31% decrease: 321.6 to 220.6 per 100,000**  33% decrease in rates for males: 298.9 to 201.6  30% decrease in rates for females: 345.6 to 240.9 | 2008-2019 | Age-standardised rates of  alcohol-related in-patient or  specialised open care (ED or  hospital admissions) per  100,000 population | YES (partial) | Reported numbers are slightly incorrect but this doesn’t change the direction or interpretation of the results. |
| Spain | Mortality attributable to alcohol in Spain | Decrease | 15-34 | 70% decrease:  Excessive drinkers: 70% decrease from 3.3 to 1.0 per 100,000  Light/Moderate drinkers: 56% decrease from 3.2 to 1.4 per 100,000 | Comparing 2001-2009 to 2010-2017 | Mortality rates attributable to  alcohol per 100,000 population | YES | N/A |
| Austria | Hospital discharges from acute hospitals | Decrease | 0-14 | 48% decrease: 280 to 145 discharges related to alcohol  51% decrease for males: 134 to 66  46% decrease for females: 146 to 79 | 2005-2019 | No. of alcohol-related hospital  discharges | YES | Denominator not known. |
| Sweden | Stafström & Raninen, 2023 | Decrease | 15-19 | Annual decrease of −7.15  (p < 0.001) for males  Annual decrease of −7.75  (p < 0.001) for  females | 2000-2021 | Linear regression of alcohol-related  harm indicators per  10,000 population | YES | N/A |
| Estonia | Alcohol market, consumption and harms in Estonia | Stable | 16-20 | Stable (7.27%): 220 to 236 turned to a doctor between timepoints | 2013-2019 | No. of patients turning to  specialists or family doctors  because of alcohol | YES | Denominator not known. |
| Lithuania | Prevalence – number of ill people | Stable | 10-19 | Stable (4.30%): 1.17 to 1.22 per 1000 | 2014-2019 | Rates of alcohol-related  diagnoses in healthcare  institutions and on death  certificates, per 1000 | YES | N/A |
| Belgium | Panken, 2023 | Unknown  (Vieira et al. report ‘Increase’ likely based on the figures) | 10-19 | Vieira et al. report increase in numbers for 10- to 19-year-olds (figures but no raw data  provided) | 2008-2019 | No. of alcohol-positive ED  admissions for entire population  in a student city | YES | No raw data, only figures. Could not access full text to re-assess figures/reproducibility and provide baseline-comparisons. |
| The Netherlands | Hospital admissions and patients | Increase | 1-20 | 21% increase: 2.4 to 2.9 per 10,000  12% increase for males: 2.6 to 2.9  22% increase for females: 2.3 to 2.8 | 2013-2019 | Rates of alcohol-related hospital  admissions per 10,000  inhabitants | YES | N/A |
| Switzerland | Patients in hospitals by age, class, sex and diagnostic group | Increase | 10-14 | 94% increase: 67 to 130 but no denominator provided | 2005-2019 | No. of alcohol-related hospital  admissions | YES | Denominator not known. |
| Switzerland | Patients in hospitals by age, class, sex and diagnostic group | Increase | 15-19 | 30% increase: 266 to 346 but no denominator provided | 2005-2019 | No. of alcohol-related hospital  admissions | YES | Denominator not known. |
| Germany | Diagnostic data of the hospitals starting from 2000 | Mixed (based on sex differences) | 10-14 | Stable (9.40% decrease): 1187 to 1076 per 100,000  Vieira et al. report the following for sex: 17% decrease for males; 31% increase for  females, concluding ‘Mixed’ | 2005-2019 | Rates of hospital discharges for  alcohol-related diagnoses per  100,000 inhabitants | YES (partial) | Cannot retrieve numbers for sex from original source but authors note issues with identifying numbers in this data. Authors only report data based on sex, but there is also data across sex which we also report here. |
| Germany | Diagnostic data of the hospitals starting from 2000 | **Increase** | 15-19 | Stable (5.50% increase): 13,624 to 14,380 per 100,000  Vieira et al. report the following for sex: 19% increase for males; 47% increase for females, concluding ‘Mixed’ because the 10-14 and 15-19 age ranges are reported together | 2005-2019 | Rates of hospital discharges for  alcohol-related diagnoses per  100 000 inhabitants | NO | **Cannot reproduce numbers for sex from original source, but authors note issues with identifying numbers in this data. Authors only report data based on sex, but there is also data across sex. There were increases for both males and females above the 10% change threshold, so this age group shows an Increase. This underscores the importance of reporting different age groups separately.** |
| Switzerland | Wicki, 2020 | Decrease  (Vieira et al. state ‘Mixed’ for age ranges combined) | 10-15 | 31% decrease:  55.1 to 37.9 per 1000 | 2010-2016 | Proportion rates of alcohol-related  hospitalisations when  accounting for Switzerland and  excluding the Canton of Vaud  (area of alcohol policy changes) | YES | Original authors categorised as ‘Mixed’ overall because 10–15-year-olds show a decrease, but 16-19 y/os show Stable trends based on the 10% threshold. Also note that this data is based on the implementation of a public health policy, which might skew the data. |
| Switzerland | Wicki, 2020 | Stable  (Vieira et al. state ‘Mixed’ for age ranges combined) | 16-19 | Stable (9.20% decrease): 53.1 to 48.2 per 1000 | 2010-2016 | Proportion rates of alcohol-related  hospitalisations when  accounting for Switzerland and  excluding the Canton of Vaud  (area of alcohol policy changes) | YES | Original authors categorised as ‘Mixed’ overall because 10–15-year-olds show decrease, but 16-19 y/os show Stable trends based on their 10% thresholds. Also note that this data is based on the implementation of a public health policy, which might skew the data. |
| Finland | Yearbook of Alcohol and Drug Statistics | **Decrease**  **(Vieira et al. state ‘Mixed’ for age ranges combined)** | <14 | 30% decrease: 329 to 244 | 2005-2019 | No. of hospital inpatient care  periods with a primary diagnosis  of alcohol | NO | **Vieira et al. state ‘Stable’ for <14-year-olds, but this is a ‘Decrease’ of 26% (they mix the results up for <14- and 15–19-year-olds).** |
| Finland | Yearbook of Alcohol and Drug Statistics | **Stable**  **(Vieira et al. state ‘Mixed’ for age ranges combined)** | 15-19 | Stable (3.85%): 260 to 250 | 2005-2019 | No. of hospital inpatient care  periods with a primary diagnosis  of alcohol | NO | **Vieira et al. state ‘Stable for <14-year-olds, but this is a ‘Decrease’ of 26% (they mix the results up for <14- and 15–19-year-olds).** |
| Mainland Europe | Danpanichkul et al., 2024 | Mixed | 15-19 | Stable APC for Finland (0.47%),  Germany (0.03%) and the Netherlands (0.22%)  Increases in APC for Belgium  (0.83%), Estonia (1.26%),  Iceland (0.92%), Lithuania  (1.54%), Norway (0.77%) and  Switzerland (1.19%) (all p <.001).  Decreases for Austria  (−0.86%), Portugal (−1.93%),  Spain (−0.72%), Sweden  (−0.57%) (all p < .001) | 2000-2019 | APC for alcohol-related cases of  chronic liver disease and  cirrhosis | YES | N/A |

**Note:** APC = Annual Percentage Change; ED = emergency department; UK = United Kingdom; US = United States. Original review reference is: Vieira E, Taylor N, Stevely A, Pennay A, Raninen J, Holmes J, et al. A systematic review of adolescent alcohol-related harm trends in high-income countries with declines in adolescent consumption. Addiction. 2025; 120: 1551-1570. https://doi.org/10.1111/add.70026

**Table S3.** Secondary data analysis of records with a comparison timepoint within the years of 2020-2022 – the years stated as the comparison in Vieira et al.’s PROSPERO registration but not adopted in their final analysis. Any changes are highlighted in **bold** in the relevant column. All references are cited in Vieira et al.

| **Country** | **First author, publication year OR data summary** | **Age** | **Comparison timepoint** | **Measure** | **Direction of trend: Vieira et al.** | **Direction of trend: re-analysis** | **Re-analysis result (“Proportion”)** | **Notes** |
| --- | --- | --- | --- | --- | --- | --- | --- | --- |
| Canada | Canadian substance use costs and harms | 0-14 | 2019 (Vieira et al.)  2020 (re-analysis) | Unstandardized rates of alcohol-attributable  ED visits per  100,000 | Decrease | Decrease | 73% decrease  60% decrease for males: 16.98 to 6.73 per 100,000  77% decrease for females: 46.71 to 10.6 per 100,000 | No change in direction. |
| Canada | Canadian substance use costs and harms | 0-14 | 2019 (Vieira et al.)  2020 (re-analysis) | Unstandardized rates of alcohol-attributable  hospitalisations per 100,000 | Stable | Stable | Stable (2% decrease):  Stable (0.91% decrease) for males: 19.73 to 19.55  Stable (1.56% increase) for females: 21.81 to 22.15 | No change in direction. |
| UK: England | Local alcohol profiles for England | <18 | 2018/2019 (Vieira et al)  2020/2021 (re-analysis) | Rates of hospital admissions  wholly attributable to alcohol  per 100,000 population | Decrease | Decrease | 56% decrease: 24,127 to 10,569 cases  60% decrease for males: 10,614 to 4218 cases  53% decrease for females: 13,513 to 6,351 | No change in direction. |
| UK: Scotland | Alcohol related hospital statistics | <15 | 2018/2019 (Vieira et al.)  2020/2021 (re-analysis) | Rates of alcohol-related hospital  admissions per 100,000  population | Decrease | Decrease | 50% decrease: 42.4 to 21.1 per 100,000  55% decrease for males: 39.3 to 17.5  46% decrease for females: 45.5 to 24.5 | No change in direction. |
| UK: Scotland | Alcohol related hospital statistics | 15-19 | 2018/2019 (Vieira et al.)  2020/2021 (re-analysis) | Rates of alcohol-related hospital  admissions per 100,000  population | Decrease | Decrease | 40% decrease  49% decrease for males: 561.8 to 284.3  30% decrease for females: 401.2 to 294.4 | No change in direction. |
| Australia | Australian alcohol-attributable harm visualisation tool | 15-34 | 2019 (Vieira et al.)  2020 (re-analysis) | Age-specific rates of alcohol-attributable  hospitalisations per 100,000 population | Mixed (based on sex differences) | Mixed | 11% decrease  16% decrease for males: 245.33 to 205.38  Stable (3.7% decrease) for females: 171.33 to 164.91 | No change in direction. |
| Sweden | Statistical database, diagnoses | 10-19 | 2019 (Vieira et al.)  2022 (re-analysis) | Age-standardised rates of  alcohol-related in-patient or  specialised open care (ED or  hospital admissions) per  100,000 population | Decrease | Decrease | 27% decrease  38% decrease for males: 298.9 to 184.8  17% decrease for females: 345.6 to 287.7 | No change in direction. |
| Austria | Hospital discharges from acute hospitals | 0-14 | 2019 (Vieira et al.)  2020 (re-analysis) | No. of alcohol-related hospital  discharges | Decrease | Decrease | 60% decrease  65% decrease for males: 134 to 47  56% decrease for females: 146 to 64 | No change in direction. |
| Estonia | Alcohol market, consumption and harms in Estonia | 16-20 | 2019 (Vieira et al.)  2021 (re-analysis) | No. of patients turning to  specialists or family doctors  because of alcohol | Stable | Stable | Stable (4.5% decrease) | No change in direction. |
| The Netherlands | Hospital admissions and patients | 1-20 | 2019 (Vieira et al.)  2021 (re-analysis) | Rates of alcohol-related hospital  admissions per 10,000  inhabitants | Increase | **Decrease** | 17% decrease  23% decrease for males: 2.6 to 2.0  17% decrease for females: 2.3 to 1.9 | **Change in direction from Increase to Decrease.** |
| Switzerland | Patients in hospitals by age, class, sex and diagnostic group | 10-14 | 2019 (Vieira et al.)  2021 (re-analysis) | No. of alcohol-related hospital  admissions | Increase | **Mixed** | 82% increase  Stable (0% change) for males: 38 to 38  190% increase for females: 29 to 84 | **Change in direction from Increase to Mixed based on sex differences.** |
| Switzerland | Patients in hospitals by age, class, sex and diagnostic group | 15-19 | 2019 (Vieira et al.)  2021 (re-analysis) | No. of alcohol-related hospital  admissions | Increase | Increase | 35% increase  39% increase for males: 163 to 227  27% increase for females: 103 to 131 | No change in direction. |

**Note:** Original review reference is: Vieira E, Taylor N, Stevely A, Pennay A, Raninen J, Holmes J, et al. A systematic review of adolescent alcohol-related harm trends in high-income countries with declines in adolescent consumption. Addiction. 2025; 120: 1551-1570. <https://doi.org/10.1111/add.70026>

**References from Pennington et al. “Table 2: Key results from a secondary data analysis focusing on late adolescence (N = 25)”.**

**Note:** We include these references in our supplementary materials rather than the main manuscript because they are already cited in Vieira et al. (2025). This avoids the issue of double citations. They are presented in the same order as Table 2.

Moise IK. Geographic gender differences in traumatic unintentional injury hospitalization and youth drinking. Drug Alcohol Depend. 2019;205:107701. <https://doi.org/10.1016/j.drugalcdep.2019.107701>

White AM, Slater ME, Ng G, Hingson R, Breslow R. Trends in alcohol-related emergency department visits in the United States: results from the nationwide emergency department sample, 2006 to 2014. Alcohol Clin Exp Res. 2018;42(2):352–9. <https://doi.org/10.1111/acer.13559>

Chen CM, Yoon Y-H. Trends in alcohol-related morbidity among community hospital discharges, united states, 2000–2015 [internet] National Institute on Alcohol Abuse and Alcoholism; 2018. Available from: <https://www.niaaa.nih.gov/sites/default/files/HDS15.pdf>

Canadian substance use costs and harms visualization tool, version 3.0.2. [Online tool] [Internet]. Canadian Substance Use Costs and Harms (CSUCH). 2023. Available from: <https://csuch.ca/explore-thedata/>

Ngo DA, Ait-Daoud N, Rege SV, Ding C, Gallion L, Davis S, et al. Differentials and trends in emergency department visits due to alcohol intoxication and co-occurring conditions among students in a U.S. public university. Drug Alcohol Depend. 2018;183:89–95. <https://doi.org/10.1016/j.drugalcdep.2017.10.029>

Smith BT, Schoer N, Sherk A, Thielman J, McKnight A, Hobin E. Trends in alcohol-attributable hospitalisations and emergency department visits by age, sex, drinking group and health condition in Ontario, Canada. Drug Alcohol Rev. 2023;42(4):926–37. <https://doi.org/10.1111/dar.13629>

Danpanichkul P, Chen VL, Tothanarungroj P, Kaewdech A, Kanjanakot Y, Fangsaard P, et al. Global epidemiology of alcohol associated liver disease in adolescents and young adults. Aliment Pharmacol Ther. 2024;60(3):378–88. <https://doi.org/10.1111/apt.18101>

Green MA, Strong M, Conway L, Maheswaran R. Trends in alcohol-related admissions to hospital by age, sex and socioeconomic deprivation in England, 2002/03 to 2013/14. BMC Public Health. 2017;17(1):412. <https://doi.org/10.1186/s12889-017-4265-0>

Sims S, Preen D, Pereira G, Fatovich D, Livingston M, O’Donnell M. Alcohol-related harm in emergency departments: linking to subsequent hospitalizations to quantify under-reporting of presentations. Addiction. 2020;116(6):1371–80. <https://doi.org/10.1111/add.15284>

Hospitalisations wholly attributable to alcohol by district 2001–2021 [Internet]. Alcohol-related harm indicators (Panga waipiro). 2021. Available from: <https://www.ehinz.ac.nz/indicators/alcohol-relatedharm/> [alcohol-related harm data]

Alcohol-related hospital statistics data [internet]. Public Health Scotland 2022. Available from: <https://publichealthscotland.scot/publications/alcohol-related-hospital-statistics/alcohol-relatedhospital-statistics-scotland-financial-year-2020-to-2021/>

Tyrrell EG, Kendrick D, Sayal K, Orton E. Poisoning substances taken by young people: a population-based cohort study. Br J Gen Pract. 2018;68(675):e703–10. <https://doi.org/10.3399/bjgp18X698897>

Australian alcohol-attributable harm visualisation tool version 2.1 [internet]. National Drug Research Institute, Curtin University and Canadian Institute for substance use Research, University of Victoria. 2023. Available from: <https://www.alcoholharmtool.info/index.php>

Alcohol-related injury: hospitalisations and deaths, 2019–20 [internet] Australian Institute of Health and Welfare; 2023. Available from: <https://www.aihw.gov.au/reports/injury/alcohol-related-injuries-2019-20/data>

Diagnoses, in-patient and/or specialised open care, alcohol index, entire Sweden, age: 10–19, both sexes [internet]. The health and welfare statistical Database 2023. Available from: <https://sdb.socialstyrelsen.se/if_par/val_eng.aspx> [statistical database, diagnoses]

Mortality attributable to alcohol in Spain, 2001–2017. (Mortalidad atribuible al alcohol en España, 2001–2017.) [Internet] Ministry of Health (Ministerio de Sanidad) Government Delegation for the National Drug Plan (Delegación del Gobierno para el Plan Nacional Sobre Drogas); 2020. Available from: <https://pnsd.sanidad.gob.es/profesionales/publicaciones/catalogo/catalogoPNSD/publicaciones/pdf/2020_Mortalidad_atribuible_al_alcohol_en_Espana_2001-2017.pdf>

Prevalence – number of ill people Institute of Hygiene (higienos institutas). 2022. Prevalence: number of ill people. Available from: <https://stat.hi.lt/default.aspx?report_id=256>

Federal Statistics Bureau (Office fédéral de la statistique). 2022. Patients in hospitals by age class, sex and diagnostic group (Patients dans les hôpitaux selon la classe l’âge, le sexe et le groupe de diagnostic) [Internet]. Available from: <https://www.bfs.admin.ch/asset/fr/px-x-1404010100_107>

Diagnostic data of the hospitals starting from 2000 . Diagnostic data of the hospitals starting from 2000 [Internet]. The Federal Health Monitoring System (Das Informationssystem der Gesundheitsberichterstattung des Bundes). 2023. Available from: <https://www.gbe-bund.de/gbe/pkg_isgbe5.prc_menu_olap?p_uid=gastd%26p_aid=38562095%26p_sprache=E%26p_help=2%26p_indnr=703%26p_indsp=3160%26p_ityp=H%26p_fid>

Wicki M, Bertholet N, Gmel G. Estimated changes in hospital admissions for alcohol intoxication after partial bans on off-premises sales of alcoholic beverages in the canton of Vaud, Switzerland: an interrupted time-series analysis. Addiction. 2020;115(8):1459–69. <https://doi.org/10.1111/add.14967>

Finnish Institute for Health and Welfare. 2006. Yearbook of Alcohol and Drug Statistics 2006 (Päihdetilastollinen vuosikirja 2006; Alkoholi ja huumeet). Finland.

Finish Institute for Health and Welfare. 2020. Yearbook of Alcohol and Drug Statistics 2020. (Päihdetilastollinen vuosikirja 2020; Alkoholi ja huumeet). Finland.
